# On the discriminatory and predictive accuracy of the RDT against the microscopy in the diagnosis of malaria among under-five children in Nigeria

**DOI:** 10.1186/s12936-019-2678-1

**Published:** 2019-02-21

**Authors:** Adeniyi Francis Fagbamigbe

**Affiliations:** 1 Department of Epidemiology and Medical Statistics, Faculty of Public Health, College of Medicine, University of Ibadan, Ibadan, Nigeria; 2 Centre for AIDS Research, Department of International Health, Bloomberg School of Public Health, Johns Hopkins University, Baltimore, USA; 3 Division of Health Sciences, Populations, Evidence and Technologies Group, Warwick Medical School, University of Warwick, Coventry, UK

**Keywords:** RDT, Microscopy, Diagnostic accuracy, Sensitivity, Specificity, NPV

## Abstract

**Background:**

Accurate identification of malaria cases is crucial to the management of cases and the eventual success of malaria eradication agenda. This study is designed to evaluate the discriminatory and predictive accuracy of malaria rapid diagnostic tests (RDTs) in Nigeria.

**Methods:**

The data obtained during the 2015 Nigeria Malaria Indicator Survey was used to quantify the discriminatory accuracy of the RDT against the microscopy through the analysis of its sensitivity, specificity, positive (LR+) and negative (LR−) likelihood ratio. The positive (PPV) and negative (NPV) predictive values, area under the receiver operating characteristic curve, and diagnostic odds ratio were used to assess the predictive accuracy of the RDTs using expert microscopy as a gold standard at p = 0.05. The McNemar paired test and the Kappa statistics were used to assess the level of agreement between the diagnostic tests.

**Results:**

There was a significant but not an excellent agreement between the results of the RDT and microscopy tests (p < 0.001). The overall sensitivity of the RDT was 87.6% (85.9–89.2%), specificity was 75.8% (74.4–77.1%), while the diagnostic accuracy stood at 79.0% (77.9–80.0%). The LR+, LR−, PPV and NPV were 3.6 (3.4–3.8) and 0.16 (0.14–0.19), 57.5% (56.1–58.9%) and 94.2% (93.5–94.9%), respectively. The sensitivity of RDT increased as the age of the children increased, from 85.7% among those aged 0–6 months to 86.1% in 7–23 month olds to 88.1% among those aged 24–59 months, but the reverse was the specificity. For children with severe anaemia, the sensitivity of the RDT was nearly 100% compared with a specificity of 39%. While the sensitivity and the PPV reduced with children’s level of anaemia, the higher the severity of anaemia, the lower the NPV, specificity, the diagnostic accuracy of the RDT. The odds of RDT being sensitive was about 50% [adjusted Odds Ratio (aOR) = 0.52 (95% CI 0.30–0.90)] lower among children aged 7–23 months compared with those aged 24–59 months while the odds of RDT being sensitive was 2 times [aOR = 2.15 (95% CI 1.67–2.77)] higher among those 7–23 months than among those aged 24–59 months.

**Conclusions:**

Although there was a significant agreement in the outcomes of RDT and microscopy tests, the discriminatory accuracy of RDT was weak. Also, the predictive accuracy, especially the PPV of the RDTS, were very low. These measures of accuracies differed across the age of the children, level of anaemia, recent experience of malaria and other characteristics. Without an accurate, efficient and reliable diagnosis of malaria, the goal of eliminating malaria and reduction of malaria-related deaths to zero by 2020 will only remain elusive.

**Electronic supplementary material:**

The online version of this article (10.1186/s12936-019-2678-1) contains supplementary material, which is available to authorized users.

## Background

Malaria remains a major public health challenge in sub-Sahara Africa, especially Nigeria [1–3]. Despite myriad efforts devoted to curbing the menace of malaria in Nigeria, its prevalence has remained high [4] with nearly half of Nigeria 20 million under-five children infected by malaria parasites. Some of the malaria eradication initiatives taken by Nigeria government and other stakeholders include mass long-lasting insecticidal net (LLIN) campaigns and distribution, replacement campaigns, intermittent preventive treatment (IPT), and a massive scale-up in malaria case management including use of RDTs.

Malaria diagnosis is a key pillar in the eradication of malaria in Africa. The World Health Organization (WHO) recommends that malaria case management is based on parasite diagnosis in all cases [5, 6] and that treatment should only commence after diagnosis [7]. The use of antigen detecting rapid diagnostic tests (RDTs) is a vital part of this strategy. Malaria RDT is a immunochromatographic lateral flow device for the detection of malaria parasite antigens [5, 8, 9]. The strategic purpose for the introduction of RDT is to extend access to malaria diagnosis by providing a parasite-based diagnosis in areas where “good-quality” microscopy cannot be maintained [5], unavailable or less convenient; hence case management is improved [10]. To enhance effective diagnosis of all malaria cases, the diagnostic method used must be accurate and available at the point of care.

Although the use of RDT has increased tremendously over the past few years and account for more than two-thirds of malaria diagnoses in Africa [11], the outcomes of such diagnoses have been a source of concern to malaria stakeholders and has threatened the success of the related sustainable development goals [12]. Recent reports on the accuracy of malaria diagnostic tests around the world are generally not satisfactory. In Nigeria, the reported 10% discrepancy (52% by RDT and 42% by microscopy) in malaria prevalence rate in 2010 Nigeria Malaria Indicator Survey (NMIS) had widened to 18% (45% by RDT and 27% by microscopy) during the 2015 NMIS. This trend may be inimical to malaria eradication in a malaria endemic setting such as Nigeria; though similar disparities have been noted elsewhere [8, 10, 13].

Literature has documented the specificities, sensitivities, numbers of false positives and false negatives and variabilities in temperature tolerances of these tests as some of the difficulties and challenges facing current RDTs [9]. Conclusive evidence is still lacking on the accuracy and safety of a test-based strategy for children [7]. It is, therefore, imperative to evaluate the performances of RDTs in terms of diagnostic accuracy as this measure provides information on the diagnostic test’s ability “discriminate between and/or predict disease and health” [14].

The consequences of erroneous and wrong diagnosis are enormous. These errors could be either false negative or false positive errors. The false negative errors occur when the disease is missed when indeed, it is present. They often result in people foregoing needed treatment and could lead to the chronic stage of disease or even death. The false positive errors are due to wrongful confirmation that a disease is present. It leads to a wrong focus on disease-free subjects which may result in unnecessary treatment and sometimes overtreatment. It could lead to a negative impact, personal inconvenience, unwarranted stress, anxiety. Eusebi et al. have already advocated for strict evaluation of the diagnostic accuracy of testing procedure aimed at validating any potential diagnostic tool [14].

This study is, therefore, designed to provide the malaria programmers with situation assessment of the accuracy of RDTs being used in Nigeria and to also determine the distribution of the levels of accuracy in terms of discrimination and prediction. It was hypothesized in this study that the outcomes of both the RDT and microscopy malaria tests are not in agreement. The findings in this study will improve programme implementation and enhance the much-needed progress towards malaria control and eradication in Nigeria.

## Methods

### Study setting

The data used for this study were collected between October and November 2015 during the 2015 NMIS that was jointly implemented by the National Malaria Elimination Programme (NMEP), the National Population Commission (NPopC), the National Bureau of Statistics (NBS), and the Malaria Partnership in Nigeria. Nigeria is a tropical country (2.66°E–14.68°E longitudes and 4.28°N–13.89°N latitudes) located at the Gulf of Guinea on the west coast Africa occupying almost a million square metres. Nigeria climates range from arid to humid equatorial. There are wide climatic variations in different parts of Nigeria. Towards the coast, temperature ranges 26–32 °C with high humidity but a much hotter temperature is prevalent in the North. Nigeria experiences a wet season from April to October with lower monthly temperatures and a dry season from November to March, with average midday temperatures of about 38 °C. Rainfall varies in Nigeria with 70 inches in the western coasts, 170 inches in the eastern coasts and 20 inches in the extreme north [15]. The diversities notwithstanding, Nigeria climate is generally very conducive for mosquitoes which are the malaria carriers. *Plasmodium falciparum* is the primary cause of malaria in Nigeria [4]. Nigeria has an annual population growth rate of 2.6% and estimated 180 million inhabitants who are greatly diversified culturally, socially and otherwise with only one-third residing in urban areas. Politically, Nigeria is divided into 36 administrative states and the Federal Capital Territory (FCT). These states were grouped into 6 regions on the basis of their location on Nigeria geographical landscape.

### Sampling

The sample design was cross-sectional and nationally representative. The most current 2006 Nigeria National Population and Housing Census were used as the sampling frame. The states were subdivided into local government areas (LGAs), with subsequent sub-divisions into localities and convenient areas, also called census enumeration areas (EAs). The EAs, which are referred to as clusters, are used as the primary sampling unit in the 2015 NMIS. There were 138 clusters in urban areas and 195 in rural areas totalling 333 clusters. A two-stage sampling strategy was used for the survey. At the first stage, 9 representative clusters were randomly selected from each of the 36 states and the FCT. In the second stage, 25 households each were randomly selected in each cluster. Thereafter, all women aged 15–49 residents in the selected households were interviewed together. Also, all children aged 6–59 months in the selected households were tested for malaria and anaemia.

### Anaemia testing

Anaemia was included in the 2015 NMIS for children aged 6–59 months as a result of the documented strong relationship between malaria infection and anaemia. With the use of a single-use retractable, spring-loaded, sterile lancet, finger/heel-prick, blood samples were drawn from every participating children and put in a microcuvette. Also, the Haemoglobin analysis was carried out on site using a battery-operated portable HemoCue^®^ analyser. The results were given to each child’s parent or guardian in both verbal and written forms within a minute of testing. Referrals for follow-up care were offered according to guidelines. The anaemia test results were recorded on the Biomarker Questionnaire, and the households counselled appropriately.

### Malaria testing using RDT

Using the same blood sample collected for anaemia testing, a drop of blood was tested immediately with the SD BIOLINE Malaria Ag P.f (HRP-II)™ (Standard Diagnostics, Inc.) RDT, being a qualitative test “to detect histidine-rich protein II antigen of *Plasmodium falciparum* in human whole blood” [4]. The test procedures were handled by well-trained field laboratory scientists in accordance with RDT manufacturer’s instructions. The RDT results were provided to each child’s parent or guardian in oral and written forms within 15 min and were recorded on the Biomarker Questionnaire. Children that tested positive to malaria and not currently on treatment with artemisinin-based combination therapy (ACT) or who had not completed a full course of the ACT during the preceding 2 weeks were given full treatment according to the Nigeria national malaria treatment guidelines [4].

### Malaria testing using blood smears

In addition to the RDT, thick and thin blood smears were prepared in the field. Each blood smear slide was labelled according to guidelines and transmitted to the laboratory. The thick and thin smear slides were stained at zonal staining and taken to the ANDI Centre of Excellence for Malaria Diagnosis, University of Lagos, Nigeria for logging and microscopic reading. Other details of the testing procedures have been reported earlier [4].

### Description of variables

The outcome variable in this study is the result of the RDT and microscopy malaria tests while the independent factors considered are child’s household wealth quintiles, child age, and sex of children, mother’s educational attainment, place of residence, region, sleeping under a long-lasting insecticide-treated net or any ever treated nets recently, experience of fever within 2 weeks preceding the survey, and the level of anaemia as used in earlier studies [3, 16]. The ages of the children were categorized into 0–6, 7–23, and 24–59 months as used in earlier studies on under-five children [17, 18].

### Data analysis

There were a total of 7011 children aged 6–59 months across all the households visited during the survey. Basic descriptive statistics were used to describe the under-five children with respect to the characteristics of their mothers. The McNemar paired test and the Kappa statistics were used to test the hypothesis of non-agreement and to determine the level of agreement between the outcomes of the diagnostic tests respectively. Diagnostic test parameters including sensitivity, specificity, positive predictive value (PPV), and negative predictive value (NPV) was used to determine the accuracy of the malaria RDT results in comparison with the “Gold standard” results from microscopy tests. The choice of microscopy as the gold standard is because it is the best available method for establishing the presence or absence of malaria. Also, the microscopy test results have been used as the gold standards in similar previous studies [7, 8, 10, 13, 16]. The Receiver Operating Curves (ROC) were used to estimate Area Under Curves (AUC).

The discriminatory accuracy of a diagnostic test is measured by its ability to correctly classify known cases (normal) and non-cases subjects (abnormal) [14]. The sensitivity, known as the True Positive Rate (TPR), is computed as proportion of True Positives (TP) among those that truly have malaria [TP + False Negative (FN)] according to the gold standard while specificity, known as the True Negative Rate (TNR), is the proportion of True Negatives (TN) among those that do not have malaria [False Positives (FP) + TN]. In contrast, the False Negative Rate (FNR) and the False Positive Rate (FPR) are (1-Sensitivity) and (1-Specificity) respectively. Generally, the higher the sensitivity and specificity, the better the RDT test.

Let *X* denote the true state of a person (microscopy test results) with microscopy positive = D^+^ and negative = D^−^. Also, let *Y* be the outcome of the RDT test, with RDT positive = T^+^ and negative = T^−^. Then,

$${\text{Sensitivity }} = P (Y = {\text{T}}^{ + } | {\text{X}} = {\text{D}}^{ + } ); \quad S{\text{pecificity}} = P(Y = {\text{T}}^{ - } |X = {\text{D}}^{ - } ) .$$

The positive likelihood ratio (LR+) and the negative likelihood ratio (LR−) are calculated as

$$LR + = \frac{TPR}{FPR} = \frac{\text{Sensitivity}}{{1 - {\text{Specificity}}}}\quad {\text{and}}\quad LR - = \frac{FNR}{TNR} = \frac{{1 - {\text{Sensitivity}}}}{\text{Specificity}}$$

as proposed by Simel et al. [19]. The unique statistics produced by the likelihood ratios have made it the optimal choice for reporting diagnostic accuracy for clinically meaningful thresholds [14].

However, there is a need to determine the predictive values since sensitivities and specificities are not measures of prediction [14]. Predictive values depend on disease prevalence, and their conclusions are transposable to other settings. The predictive values help to determine how likely the disease is, given the test result. The PPV is the probability that the disease is present, given that the diagnostic test is positive. It is computed as TP/(TP + FP) while the NPV is the probability that the disease is not present given that the test is negative, computed as TN/(TN + FN). A diagnostic test could be said to be perfect if it can predict perfectly, i.e., if PPV = NPV = 1. The PPV decreases with decreasing prevalence.

$${\text{PPV}} = P ( {\text{D}}^{ + } | {\text{T}}^{ + } );\quad {\text{NPV}} = P ( {\text{D}}^{ - } | {\text{T}}^{ - } ).$$

The accuracy of a test = (TP + TN)/(TP + TN + FP + FN) and its confidence intervals are the standard logits as given by Mercaldo et al. [20]. The pretest probability is the same as the prevalence as determined by the gold standard, the pretest odds is prevalence/(1 − prevalence), posttest odds = pretest odds * likelihood ratio and the posttest probability = posttest odds/(1 + positive odds).

The ROC analysis is used in diagnostic screening evaluation to quantify the accuracy of diagnostic tests [21]. The “roccomp” and “rocgold” implemented as ado-files Stata version 12 were used to plot the ROCs and compare the ROCs among different categories of children’s characteristics. The Lorenz curve, a measure of inequality and can be inscribed in the area between the curve and the diagonal line, was computed and quantified by the Gini index and the Pietra index. In all, data were weighted, the significance level was set at 5% with confidence intervals were estimated using earlier proposed methods [19, 22].

## Results

Among the 7011 children included in the survey, only 6025 and 5753 children were tested for malaria parasite using the microscopy and the RDT methods respectively. The distribution of the results of the tests by selected children characteristics is presented in Table 1. For the two test procedures, malaria prevalence increased with the children ages and was insignificantly higher among male children than among female children. In all, children residing in the rural areas, Northern geopolitical zones, who came from homes in the poorer wealth quintiles and who slept under treated nets recently and who had fever within the 2 weeks preceding the survey had a higher significant prevalence of malaria for both the RDT and microscopy malaria tests.

**Table 1:** Distribution of the results of the malaria tests using RDT and microscopy and their association with selected children characteristics in the Nigeria 2015 MIS

Children Characteristic	Number (n = 7011)	%	Malaria test results			
			RDT+ (%)	*x*^2^ p-value	Micro+ (%)	*x*^2^ p-value
Child’s age (months)						
0–6	755	10.8	21.6	< 0.001*	10.0	< 0.001*
7–23	1914	27.3	36.2		20.3	
24–59	4342	61.9	49.5		30.9	
Sex						
Male	3574	51.0	46.2	0.063	27.8	0.276
Female	3437	49.0	44.0		26.9	
Location						
Urban	2350	33.5	24.1	< 0.001*	11.4	< 0.001*
Rural	4661	66.5	55.7		35.6	
Zone						
North Central	1309	18.7	50.7	< 0.001*	32.1	< 0.001*
North East	983	14.0	42.9		26.5	
North West	2286	32.6	58.2		37.1	
South East	601	8.6	31.7		13.9	
South South	780	11.1	28.9		19.4	
South West	1053	15.0	32.1		15.3	
Usual resident						
No	69	1.0	36.8	0.147	25.2	0.598
Yes	6942	99.0	45.2		27.4	
Wealth quintile						
Poorest	1474	21.0	64.1	< 0.001*	43.1	< 0.001*
Poorer	1612	23.0	62.7		41.0	
Middle	1333	19.0	49.2		27.7	
Richer	1289	18.4	30.2		16.8	
Richest	1303	18.6	12.7		4.3	
Slept under ever treated net						
No	3963	56.5	42.1	< 0.001*	25.7	< 0.001*
Yes	3048	43.5	49.0		29.5	
Slept under LLITNs						
No	3980	56.8	42.0	< 0.001*	25.7	< 0.001*
Yes	3031	43.2	49.1		29.5	
Fever in last 2 weeks						
No	4123	59.0	37.4	< 0.001*	23.7	< 0.001*
Yes	2868	41.0	55.1		32.1	
Anaemia level						
Severe	224	3.7	87.6	< 0.001*	67.4	< 0.001*
Moderate	2376	39.4	61.9		40.6	
Mild	1524	25.3	37.5		20.6	
Not anaemic	1905	31.6	25.1		12.9	
Total	7011	100.0	41.5		27.3	

*RDT+* positive on the rapid diagnostic test, *Micro+* positive on microscopy test

* Significant at 5% x^2^ test

In addition, the level of anaemia was significantly associated with the presence of malaria parasite as evidenced by both the RDT and microscopic tests. Children with severe anaemia had higher malaria prevalence (RDT+ = 88%; microscopy+ = 67%) compared with others that were not anaemic (RDT+ = 25%; microscopy+ = 13%). Although not stated in the tables, *P. falciparum* was the main (94%) type of malaria parasite found through the blood film microscopy among the U5 children, followed by the *Plasmodium ovale* and *Plasmodium malariae* at 5% and 1%, respectively.

The outcome of the McNemar paired test used in testing the hypothesis of non-agreement between the RDT and microscopy tests showed evidence of a significant agreement between the two tests with test statistics of 553.47 with p < 0.0001 while the Kappa statistics of the agreement was 0.55.

Based on the microscopy test as a gold standard, Table 2 shows the discriminatory and predictive accuracies of the RDT test by the characteristics of the 5753 children that had both tests. The overall sensitivity was 88%, specificity was 76% while the diagnostic accuracy (that is the overall agreement) of RDT stood at 79%. The overall positive and negative likelihood ratio was 3.6 and 0.16 respectively while the overall PPV and NPV were 58% and 94%, respectively. The sensitivity of RDT increased significantly as the age of the children increased (p < 0.05), from 86% among those aged 0–6 months to 86.1% in 7–23 month olds to 88.1% among those aged 24–59 months. The reverse was the specificity, LR+, and the NPV but diagnostic accuracy of RDT and the PPV increased with the age of the children (p < 0.05). On the sex of the children, there was no difference in the sensitivity of RDT but the specificity differed with significantly higher proportions among females (77%) than males (75%) (p < 0.05). Interestingly, RDT was more sensitive in the rural area at 88% than in urban areas (84%) but less specific in rural (68%) than in the (86%) (p < 0.05).

**Table 2:** The distribution of diagnostic discriminatory accuracy, likelihood ratios and the predictive values of RDT against microscopy test by selected children characteristics in the Nigeria 2015 MIS

Characteristics	Sensitivity	Specificity	Total agreement	LR+	LR−	PPV	NPV
	% (95% CI)	% (95% CI)	% (95% CI)	LR+ (95% CI)	LR− (95% CI)	% (95% CI)	% (95% CI)
Total	87.6 (85.9–89.2)	75.8 (74.4–77.1)	79.0 (77.9–80.0)	3.6 (3.4–3.8)	0.16 (0.14–0.19)	57.5 (56.1–58.9)	94.2 (93.5–94.9)
Children age							
0–6 Months	***85.7 (42.0***–***99.3)***	***90.1 (50.1***–***95.6)***	***89.7 (83.0***–***96.5)***	***8.7 (4.0***–***18.7)***	0.16 (0.03–0.97)	***46.1 (20.4***–***73.8)***	***98.5 (90.5***–***99.9)***
7–23 Months	***86.1 (81.9***–***89.3)***	***80.7 (78.6***–***82.7)***	***81.8 (80.0***–***83.6)***	***4.5 (4.0***–***5.0)***	0.17 (0.13–0.22)	***53.3 (59.1***–***57.4)***	***95.8 (94.4***–***96.8)***
24–59 Months	***88.1 (86.0***–***89.8)***	***72.9 (71.1***–***74.5)***	***77.5 (76.2***–***78.8)***	***3.2 (3.0***–***3.5)***	0.16 (0.14–0.19)	***58.9 (56.6***–***61.2)***	***93.2 (92.0***–***94.2)***
Sex							
Male	87.6 (85.1–89.8)	***74.7 (72.8***–***76.6)***	78.3 (76.7–79.8)	3.5 (3.2–3.7)	0.17 (0.14–0.20)	57.2 (55.3–59.1)	93.9 (92.8–94.9)
Female	87.6 (85.0–89.9)	***76.9 (75.0***–***78.7)***	79.7 (78.2–81.2)	3.8 (3.5–4.1)	0.16 (0.13–0.20)	57.8 (55.7–59.8)	94.5 (93.4–95.4)
Location							
Urban	***83.6 (78.3***–***88.1)***	***86.3 (84.6***–***87.9)***	***86.0 (96.7***–***98.1)***	***6.1 (5.4***–***7.0)***	0.19 (0.14–0.25)	***45.5 (42.3***–***48.8)***	***97.5 (96.7***–***98.1)***
Rural	***88.3 (86.5***–***90.0)***	***68.3 (66.4***–***70.1)***	***75.3 (72.9***–***76.7)***	***2.8 (2.6***–***3.0)***	0.17 (0.15–0.20)	***60.2 (58.7***–***61.7)***	***91.5 (90.2***–***92.6)***
Zone							
North Central	***92.8 (89.5***–***95.3)***	***73.5 (70.4***–***76.5)***	***79.1 (76.6***–***81.4)***	***3.5 (3.1***–***3.9)***	***0.10 (0.07***–***0.14)***	***58.5 (55.7***–***61.3)***	***96.2 (94.5***–***97.4)***
North East	***86.7 (82.3***–***90.4)***	***74.3 (71.2***–***77.3)***	***77.6 (75.0***–***80.0)***	***3.4 (3.0***–***3.8)***	***0.18 (0.13***–***0.24)***	***54.5 (51.4***–***57.7)***	***94.0 (92.1***–***95.5)***
North West	***85.0 (81.9***–***87.8)***	***63.3 (59.9***–***66.6)***	***72.2 (69.8***–***74.5)***	***2.3 (2.1***–***2.6)***	***0.24 (0.19***–***0.29)***	***61.5 (59.3***–***63.7)***	***86.0 (83.4***–***88.2)***
South East	***94.0 (86.5***–***98.0)***	***80.6 (76.8***–***84.4)***	***82.6 (79.2***–***85.6)***	***4.9 (4.0***–***5.9)***	***0.07 (0.03***–***0.17)***	***45.4 (40.7***–***50.1)***	***98.7 (97.1***–***99.5)***
South South	***79.5 (72.1***–***85.6)***	***85.4 (82.3***–***88.1)***	***84.2 (81.4***–***86.8)***	***5.5 (4.4***–***6.7)***	***0.24 (0.18***–***0.33)***	***58.3 (53.0***–***63.3)***	***94.2 (92.2***–***95.7)***
South West	***93.4 (87.5***–***97.1)***	***84.9 (81.8***–***87.7)***	***86.3 (83.6***–***88.7)***	***6.2 (5.1***–***7.5)***	***0.08 (0.04***–***0.15)***	***55.3 (50.5***–***60.1)***	***98.5 (97.1***–***99.2)***
Fever in the last 2 weeks							
No	86.5 (83.8–88.9)	***82.7 (81.2***–***84.2)***	***83.6 (82.3***–***84.9)***	***5.0 (4.6***–***5.5)***	***0.16 (0.14***–***0.20)***	***60.1 (57.9***–***62.3)***	95.3 (94.4–96.1)
Yes	88.6 (86.2–90.7)	***65.8 (63.5***–***68.1)***	***73.2 (71.4***–***74.9)***	***2.6 (2.4***–***2.8)***	***0.17 (0.14***–***0.21)***	***55.4 (53.6***–***57.1)***	92.4 (90.9–93.6)
Treated fever							
No	***92.8 (87.7***–***96.4)***	***70.5 (63.1***–***77.2)***	***81.1 (76.4***–***85.2)***	***3.2 (2.5***–***4.0)***	***0.10 (0.06***–***0.18)***	***73.9 (69.1***–***78.1)***	91.7 (86.2–95.2)
Yes	***87.6 (84.4***–***90.0)***	***65.3 (62.9***–***67.7)***	***92.4 (90.9***–***93.7)***	***2.5 (2.3***–***2.7)***	***0.19 (0.15***–***0.23)***	***52.2 (50.3***–***54.0)***	92.4 (90.9–93.7)
Slept under LLITNs							
No	86.7 (84.3–88.9)	***78.0 (76.3***–***79.6)***	***80.3 (78.9***–***81.6)***	***3.9 (3.6***–***4.3)***	0.17 (0.14–0.20)	57.7 (54.9–60.3)	94.5 (93.4–95.4)
Yes	88.6 (85.9–90.8)	***72.5 (70.2***–***74.6)***	***77.2 (75.5***–***78.9)***	***3.2 (2.9***–***3.5)***	0.16 (0.13–0.15)	57.4 (54.4–60.3)	93.8 (92.4–95.1)
Building material							
Totally improved	***87.8 (84.2***–***90.6)***	***82.5 (80.8***–***84.1)***	***83.4 (82.0***–***84.8)***	***5.0 (4.6***–***5.5)***	***0.14 (0.11***–***0.19)***	***49.9 (46.3***–***53.5)***	***97.1 (96.3***–***97.8)***
Partially improved	***89.2 (86.5***–***91.5)***	***71.1 (68.7***–***73.3)***	***76.6 (74.8***–***78.4)***	***3.1 (2.8***–***3.4)***	***0.15 (0.12***–***0.19)***	***57.5 (54.4***–***60.6)***	***93.7 (92.1***–***95.1)***
Nothing improved	***85.1 (81.5***–***88.2)***	***60.4 (55.9***–***64.7)***	***72.4 (69.6***–***75.3)***	***2.2 (1.9***–***2.4)***	***0.24 (0.20***–***0.31)***	***67.1 (63.1***–***70.8)***	***81.1 (76.9***–***84.9)***
Anaemia level							
Severe	***97.7 (93.5***–***99.5)***	***38.7 (26.6***–***51.9)***	***78.8 (72.3***–***84.3)***	***1.6 (1.3***–***2.0)***	***0.06 (0.02***–***0.19)***	***77.1 (73.4***–***80.4)***	***88.9 (71.5***–***96.2)***
Moderate	***89.3 (87.1***–***91.3)***	***63.7 (61.0***–***66.3)***	***74.2 (72.3***–***76.0)***	***2.5 (2.3***–***2.7)***	***0.17 (0.14***–***0.20)***	***63.0 (61.2***–***64.8)***	***89.6 (87.7***–***91.3)***
Mild	***82.7 (78.0***–***86.8)***	***77.7 (75.2***–***80.0)***	***78.7 (76.6***–***80.8)***	***3.7 (3.3***–***4.2)***	***0.22 (0.17***–***0.28)***	***49.5 (46.6***–***52.5)***	***94.4 (93.0***–***95.6)***
Not anaemic	***81.9 (76.5***–***86.5)***	***85.1 (83.3***–***86.8)***	***84.7 (83.0***–***86.3)***	***5.5 (4.8***–***6.3)***	***0.21 (0.16***–***0.28)***	***44.3 (41.2***–***47.5)***	***97.0 (96.1*** **-** ***97.7)***

*Proportions in Bold and Italics* are significantly different at 5% significance level

Also, there was a significant PPV gap of 14% in a rural area (60%) compared with the urban area (46%) (p < 0.05) but with a closer NPV (92% vs 98%) (p < 0.05). The overall accuracy, the sensitivity, the specificity, LR+, and NPV of the RDT were generally and significantly higher across the southern regions in Nigeria than in the northern regions (p < 0.05). For children with severe anaemia, the sensitivity of the RDT was nearly 100% compared with a specificity of 39% (p < 0.05). It appeared that the discriminatory accuracy reduced with children level of anaemia but the higher the severity of the anaemia, the lower its specificity (p < 0.05). However, both the LR+ and the LR− increased as the severity of anaemia increased (p < 0.05). For the predictive accuracy, PPV reduced with reduced severity from 77 to 44% (p < 0.05) among children that didn’t have anaemia while the NPV increased significantly from 90% among children that had severe anaemia to 97% among those that didn’t have anaemia (p < 0.05) as shown in Table 2.

**Fig. 1:**
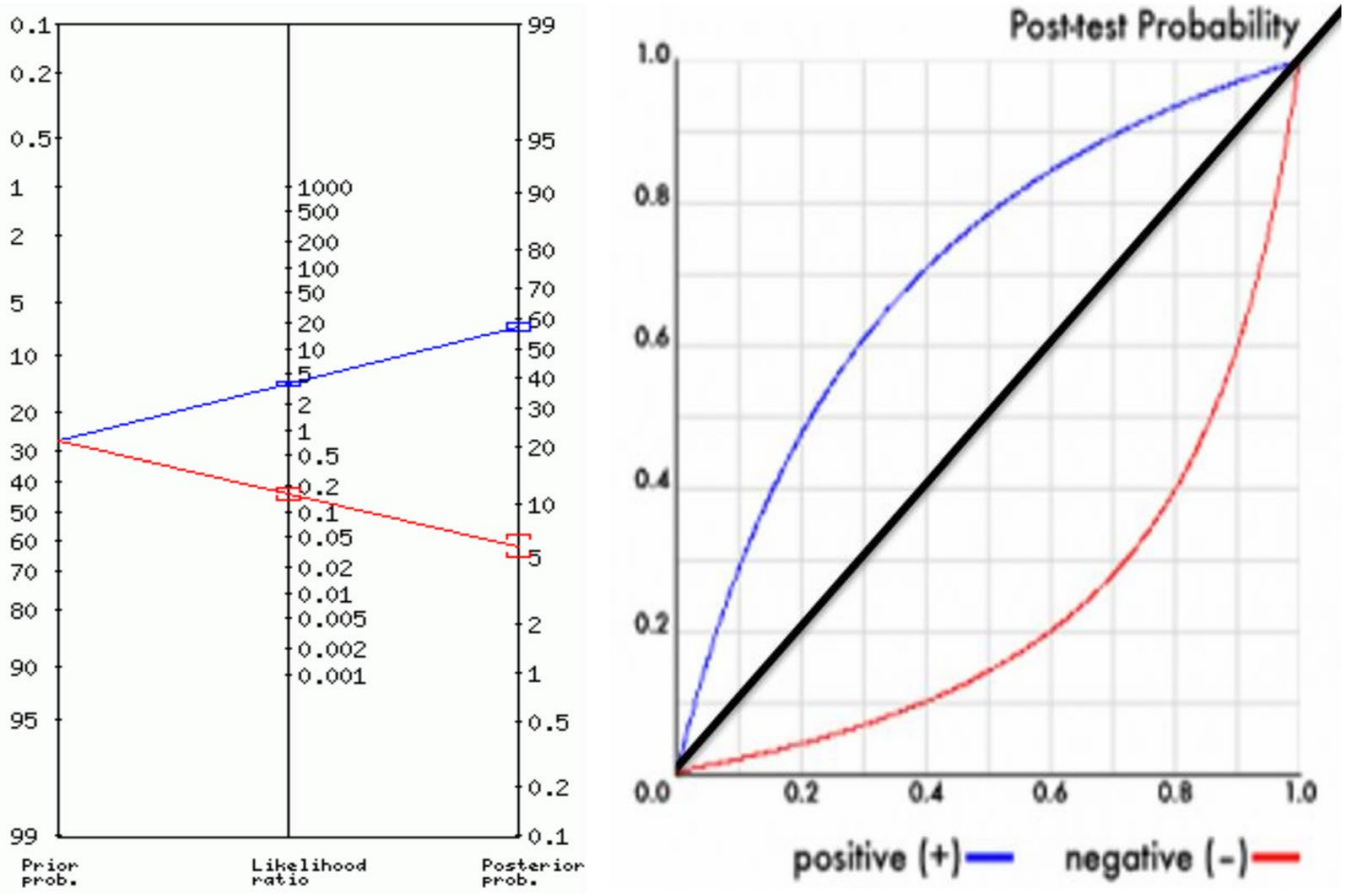
The prior probability, likelihood ratio and the posterior probability of malaria among under-five children in Nigeria

Among all the under-five children, the prior probability of malaria was 27%. For the positive tests (showed in blue line in Fig. 1), the LR+ is 3.62 (95% CI 3.42–3.83), the posterior probability (odds) is 58% (95% CI 56–59%) and approximately 1 in 1.7 with positive test results actually had the parasite while approximately 1 in 1.1 who tested negative was actually negative, LR− is 0.16 (95% CI 0.14–0.19) with corresponding posterior probability (odds) of 0.06 (95% CI 0.05–0.07) as shown in Fig. 1.

**Fig. 2:**
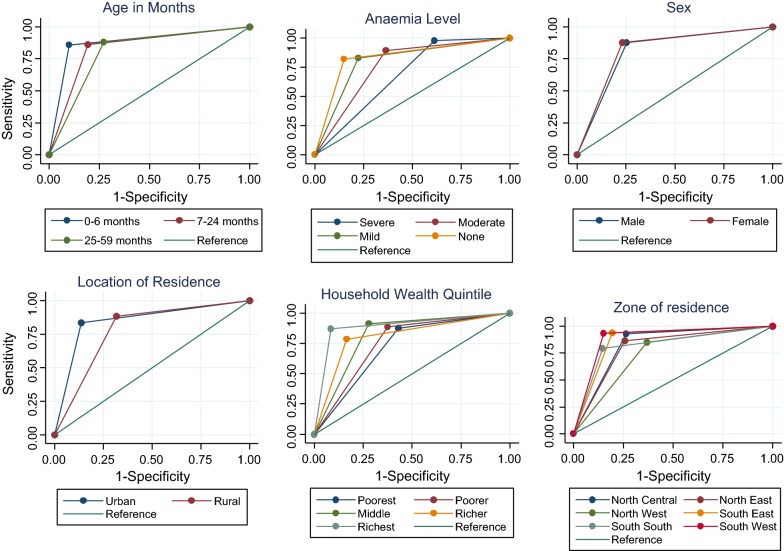
The ROC curve of the accuracy of the RDT test by the selected characteristic of under five children in Nigeria 2015 MIS

As shown in Table 3, the AUC of the correct diagnosis by the RDT in comparison with the microscopy test by selected children characteristics was 82% which suggests that 4 of every 5 children were correctly classified by the RDT. For the total AUC, the Gini index and Pietra index for the Lorenz curve were both equal to 0.6337 which is the level of agreement between the RDT and the microscopy test. The AUC among children aged 0–6 months was 88% compared with 83% and 80% among those aged 7–23 and 24–59 months respectively (p < 0.05). On wealth status, the AUC ranged from 89 to 72% for children from richest and poorest households respectively (p < 0.05). On the level of anaemia, AUC was 68% for those that had severe anaemia, 77% for moderate anaemia, 80% for mild anaemia and 84% for those with no anaemia (p < 0.05). The AUCs were significantly different across the age of the children, the zone and location of their residence, their household wealth quintile, mothers’ education, quality of housing material, haven had fever within 2 weeks preceding the data collection as well as the level of anaemia (p < 0.05). The ROC and Lorenz curves for all children are shown in Additional file 1, while the ROC curves for some selected children characteristics are shown in Fig. 2.

**Table 3:** The distribution of area under curve by selected children characteristics in the Nigeria 2015 MIS

Characteristics	AUC (95% CI)	X^2^	p = value
Children age			
0–6 months	87.9 (73.5–100)	6.63	0.04*
7–23 months	83.4 (81.3–85.5)		
24–59 months	80.4 (79.2–81.7)		
Sex			
Male	81.2 (79.7–82.6)	1.02	0.31
Female	82.2 (80.7–83.7)		
Zone			
North Central	83.2 (81.1–85.2)	92.13	0.00*
North East	80.5 (78.0–83.0)		
North West	74.2 (72.0–76.3)		
South East	87.3 (84.2–90.4)		
South South	82.5 (78.9-86.0)		
South West	89.2 (86.6–91.8)		
Location			
Urban	85.0 (82.5–87.5)	21.98	0.00*
Rural	78.3 (77.0–79.6)		
Mother education			
Education	76.3 (74.6–77.9)	38.03	0.00*
Primary	81.9 (79.3–84.5)		
Secondary	84.0 (81.6–86.4)		
Higher	90.6 (82.5–98.5)		
Wealth quintile			
Poorest	72.3 (69.8–74.8)	61.93	0.00*
Poorer	75.6 (73.4–77.9)		
Middle	81.8 (79.6–83.9)		
Richer	80.8 (77.7–83.9)		
Richest	89.3 (84.7–93.9)		
Building materials			
Totally improved	85.2 (83.4–86.9)	58.89	0.00*
Partially improved	80.1 (78.5–81.8)		
Nothing improved	72.8 (70.1–75.5)		
Had fever recently**			
No	84.6 (83.2–86.1)	46.61	0.00*
Yes	77.2 (75.7–78.8)		
Anaemia level			
Severe	68.2 (62.0–74.5)	32.25	0.00*
Moderate	76.5 (74.8–78.2)		
Mild	80.2 (77.8–82.6)		
Not anaemic	83.5 (80.9–86.1)		
Total	81.7 (80.6–82.7)		

* Significant at 5%

** Within 2 weeks preceding the survey Significant at 5% Chi square test

The adjusted odds of RDT being sensitive was about 50% (aOR = 0.52; 95% CI 0.30–0.90; p < 0.05) lower among children aged 7–23 months compared with those aged 24–59 months. The adjusted odds of RDT being sensitive was 11 times (aOR = 11.30; 95% CI 2.58–49.60; p < 0.05) and 2 times (aOR = 2.15; 95% CI 1.67–2.77; p < 0.05) higher among those aged 0–6 months and 7–23 months respectively than among those aged 24–59 months. Also, the adjusted odds of RDT being sensitive was significantly over five times more likely among children with severe anaemia than those with moderate anaemia, while the odds of being specific was about three times higher among children with no anaemia than children with moderate anaemia (p < 0.05) (see Table 4).

**Table 4:** Adjusted factors influencing the sensitivity, specificity and diagnostic accuracy of the RDT compared with the microscopy in Nigeria 2015 MIS

Characteristics	Sensitivity		Specificity		Diagnostic accuracy	
	aOR (95% CI)	p-value	aOR (95% CI)	p-value	aOR (95% CI)	p-value
Child age (24–59 months)						
0–6 months	0.53 (0.04–6.84)	0.63	11.3 (2.58–49.6)*	0.00	3.88 (1.16–12.9)*	0.03
7–23 months	0.52 (0.30–0.90)*	0.02	2.15 (1.67–2.77)*	0.00	1.32 (1.07–1.62)*	0.01
Anaemia level (moderate)						
Severe	5.46 (1.27–23.4)*	0.02	0.61 (0.30–1.24)	0.17	1.84 (1.16–2.94)*	0.01
Mild	0.80 (0.41–1.56)	0.52	1.80 (1.36–2.40)*	0.00	1.13 (0.89–1.44)	0.31
Not anaemic	0.38 (0.19–0.76)*	0.01	2.88 (2.15–3.86)*	0.00	1.45 (1.13–1.86)*	0.00
Treated malaria (yes)**						
No	1.73 (1.26–2.38)*	0.00	1.53 (0.76–3.08)	0.23	1.33 (0.90–1.98)	0.16
Zone (North west)						
North Central	6.49 (1.84–22.8)*	0.00	0.80 (0.55–1.17)	0.25	0.95 (0.69–1.30)	0.74
North East	1.31 (0.69–2.52)	0.41	1.15 (0.83–1.59)	0.39	1.02 (0.78–1.32)	0.89
South East	2.52 (0.6–10.55)	0.21	0.87 (0.53–1.41)	0.57	1.0 0(0.66–1.52)	1.00
South South	1.45 (0.53–3.95)	0.47	1.30 (0.79–2.13)	0.30	1.21 (0.80–1.83)	0.36
South West	3.29 (0.82–13.25)	0.09	0.78 (0.44–1.35)	0.37	0.87 (0.54–1.40)	0.58
Location (Rural)						
Urban	0.54 (0.23–1.27)	0.16	1.24 (0.90–1.70)	0.19	0.99 (0.74–1.31)	0.92
Sex (Male)						
Female	1.40 (0.86–2.30)	0.18	1.01 (0.81–1.28)	0.91	1.06 (0.87–1.28)	0.58
Wealth quintile (poorest)						
Poorer	0.73 (0.38–1.40)	0.34	1.22 (0.85–1.74)	0.28	1.21 (0.91–1.59)	0.19
Middle	1.24 (0.42–3.68)	0.70	1.65 (1.08–2.53)*	0.02	1.42 (1.01–2.03)*	0.04
Richer	0.34 (0.10–1.13)	0.08	2.31 (1.37–3.91)*	0.00	1.49 (0.95–2.32)	0.08
Richest	0.53 (0.10–2.73)	0.44	3.04 (1.61–5.71)*	0.00	2.19 (1.26–3.83)*	0.01

*Significant at 5%

**Within 2 weeks preceding the survey

## Discussion

This study was designed to evaluate the discriminatory and predictive accuracy of the RDT malaria test against the microscopy smear test as the “gold” standard. There were significant relationship and agreement between the outcomes of the RDT and microscopy malaria test procedures. However, the Kappa statistics at 0.55 and the Gini and Pietra indexes both at 0.6337 showed that the agreement was only good but not excellent. Generally, the RDT was found to be more sensitive (88%) than being specific (76%). The sensitivity in the current study is comparable to the sensitivity of 89% but lower than the specificity of 88% reported in an earlier longitudinal study in Tanzania [13]. In the same report, 63% sensitivity and 94% specificity were obtained in the cross-sectional component of the study. The authors noted that these measurements of discriminatory analysis varied widely and it depended on the presence of fever and the parasite density [13].

Murungi et al. noted that poor specificity in a diagnostic test may negatively impact RDT-based diagnostic strategies for malaria [23]. The wide disparities in the performance of RDTs against the microscopy and the PCR motivated Murungi et al. to explore the accuracy of the HRP2 and pLDH RDTs and the microscopy in a two-step algorithm among 276 individuals. The authors found varying and very high levels of sensitivity and specificity depending on the stage of malaria. They concluded that certain RDTs could be more accurate in new cases and initial diagnosis than in malaria case monitoring and treatment and vice versa [23].

On the predictive accuracy of the RDTs used in malaria diagnosis in the current study, the PPV was low at 58% while the NPV was relatively high at 94%, respectively. This finding suggests that the likelihood of having malaria when the RDT test is positive is only about half while the likelihood of being disease-free when the RDT test is negative is very high. In contrast, Hopkins et al. found that the PPV of the HRP2-based test was 98% compared with “the expert” microscopy, with an NPV of 97% for the HRP2-based test [8]. Also, a study carried out in Burkina Faso found the PPV and NPV of RDT to be 9% and 99.8%, respectively, in the dry season compared with 82% and 84% in the rainy season for infants to over 99% for adults [7]. However, it is worth noting that PPVs are functions of disease prevalence.

In the current study, the sensitivity of RDT increased as the age of the children increased, while the specificity reduced. This is similar to the findings of Nankabinwa et al. that the sensitivity and specificity of PCR and RDTs varied by age of the study participants. While the sensitivity of the RDT was similar for both males and females, the specificity was only slightly higher among females than males [16]. All the diagnostics accuracy examined in the current study varied by both the rural–urban differentials and the region of location of the place of residence of the children. These indices were generally higher across the urban areas and in the Southern regions in Nigeria. Similar findings have been reported in an earlier study where it was noted that the sensitivity and specificity of microscopy and RDT against the PCR varied across the study sites [16].

Also, the level of anaemia influenced both the discriminatory and the predictive accuracies of the RDTs. The RDTs were totally sensitive and less than 40% specific among children with severe anaemia. The higher the severity of anaemia in children, the higher the sensitivity and the lower the specificity. In a similar trend, PPV reduced with reducing anaemia severity, while the NPV increased with a reduction in the level of severity of anaemia.

Although, all the tests in the current study were carried out during the same dry season which eliminated seasonal variability. It cannot be ascertained in the current study if the dry season influenced the accuracies of the RDTs in a study conducted in Burkina Faso. Bisofi et al. had found a significant effect of seasonality in the discriminatory accuracy of RDT. It was reported that while the sensitivity and specificity of the RDT were 86% and 90% respectively in the dry season, the figures were 94% and 78% respectively in the rainy season [7]. The same study found seasonal variability in PPV and NPV of RDT to be 9% and 99.8% respectively in the dry season, compared with 82% and 84% respectively in the rainy season among infants [7]. In addition, Mouatcho et al. found that the specificities, sensitivities, numbers of false positives, numbers of false negatives and temperature tolerances of the RDTs vary considerably and are some of the challenges facing the accuracy of RDTs [9]. Other factors that may influence diagnostic accuracies are the efficiency of RDT storage, transport or handling of malaria RDTs as well as the expertise of the handlers [5], but these factors are not available for assessment in the current study.

Malaria RDTs are generally designed to be used in malaria-endemic areas where good-quality microscopies are out of reach. It is a requirement that a diagnostic method must be accurate and available at the point of care if the effective diagnosis of all malaria cases is to be achieved. A diagnostic test with a high degree of sensitivity usually has a low false negative rate which ensures that only a few true cases are not correctly classified. It is, therefore, imperative that a screening test used in ruling out cases should have a reasonably high degree of sensitivity. In the same vein, a diagnostic test with a high degree of specificity produces low false positive rates which invariably leads to only a few misdiagnosed subjects. A confirmatory test used in ruling-in cases should have a high degree of specificity.

There is a need to adhere to the WHO recommendations on the procurement of malaria RDTs [5] as well as on the storage and use of malaria RDTs. Total adherence to these recommendations will substantially improve the discriminatory and predictive accuracy of the malaria RDTS. According to the WHO, to be reliable, “RDTs should be sensitive enough to reliably detect malaria parasites at densities associated with disease” [24]. This suggests that sensitivity of an RDT is a function of the quality of manufacture, species, number, viability, and strain of parasites present, RDT conditions, storage conditions, application technique and level of care exercised. The level of concentration of target antigen present as well as the level of parasite density has much influence on the accuracies of RDTs. Nonetheless, both the sensitivity and specificity of an RDT must be high, so that both malaria and non-malarial fevers are correctly captured and thereby given adequate management, although higher sensitivity is preferable to high specificity so as to reduce deaths that could arise from a missed parasitaemia [24]. The WHO recommendation stated further that choice of RDT must be guided by the panel detection score (PDS) against the *P. falciparum* and against the *P. vivax*, which must be at least 75% in both cases and that the false positive rates and invalid rate should be less than 10% and 5%, respectively [5]. For RDTs to perform optimally, there must be a demonstration of the presence of parasitaemia, efficient mechanism for quality control, “cool chain” for transport and storage, adequately trained health worker and, adequate monitoring [5].

## Conclusions

This study found that the discriminatory accuracy of RDT used during the 2015 MIS survey was not strong enough. Also, the predictive accuracy, especially the positive predictive value of the RDTS, were low although the quality for RDT and microscopy used during the survey were not assessed in this study. These measures of accuracies differed across the age of the children, level of anaemia, recent experience of fever, rural–urban as well as geographical differences in the location of residence. The discriminative and predictive accuracy of a diagnostic test is a function of both the clinical diagnostic pathway and the costs of outcome misclassification. There is a need to improve on the accuracy of RDTs among this vulnerable population sub-group although the PPVs are functions of the malaria prevalence in the studied population.

### Recommendation

Despite the challenges and inadequacies in the accuracies of RDTs in both the ability to discriminate positive and negative cases and to also predict who actually has malaria, the RDTs remained the best option especially in areas with limited access to microscopy. Therefore, there is a need to improve the performance of the RDTS. Given the wide gap between the malaria prevalence from the two procedures used in the 2015 MIS, the weak discriminatory accuracy and the rather weaker predictive accuracy of the RDTs and the fact that RDTs play important role in the improvement of malaria diagnoses and consequent management of malaria, it is recommended that two different sets of RDTs should be used in the field. Similar advocacy has been made earlier in other settings [10]. The authors submitted that two RDTs should be used together because they individually had superior sensitivity and superior specificity respectively [10]. In addition, Murungi et al. confirmed that the use of a two-step malaria diagnostic algorithm wherein the microscopy served as a confirmatory test for indeterminate HRP2+/pLDH− has a significantly better specificity with a similar level of sensitivity [23].

### Strength and limitations

Due to the secondary data used in this study, the choices of explanatory variables were limited. The author could not assess the quality of the RDT and microscopy test used in the survey due to logistic constraints. The levels of the quality of the test procedures might have affected the diagnosis outcomes and the results. Although the report of the MIS study design stated that the microscopy handlers were experts who were well trained, their performances may differ and affect the microscopy test results. Also, the current study cannot ascertain whether molecular methods were used during the MIS. Regardless of these limitations, the use of nationally representative household data for this analysis makes the findings in this study generalizable in Nigeria. This is supported by an assertion that “household surveys are important tools for monitoring the malaria disease burden and measuring the impact of malaria control interventions with parasite prevalence as the primary metric” [16]. Also, the testing procedure was verified on a reasonable population which was made up of people with varying degree of malaria severity thereby giving credence to the reliability as previously advocated [14]. The study has provided an evidence-based assessment of the accuracy and reliability of RDTs used for diagnoses of malaria among under-five children in Nigeria to assist malaria and child health programmers.

## Additional file

**Additional file 1.** The ROC and Lorenz curve of the accuracy of RDT test against the Microscopy test in the Nigeria 2015 MIS.
