# Supplementary figures and images for: On the discriminatory and predictive accuracy of the RDT against the microscopy in the diagnosis of malaria among under-five children in Nigeria
Source: Malar J. 2019 Feb 21;18:46. doi: 10.1186/s12936-019-2678-1 (PMC6385408; doi:10.1186/s12936-019-2678-1)

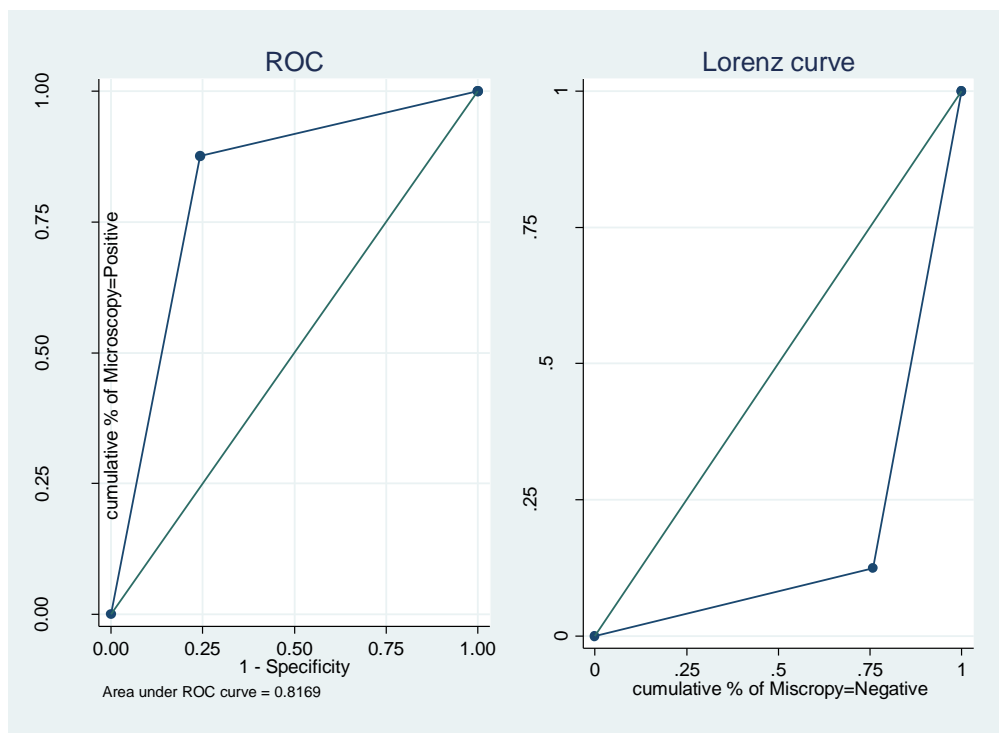

Supplement: Supplementary file 1 — Additional file 1. The ROC and Lorenz curve of the accuracy of RDT test against the Microscopy test in the Nigeria 2015 MIS. [file 12936_2019_2678_MOESM1_ESM.pdf]
